# High Prevalence of Asthenopia among a Population of University Students

**DOI:** 10.18502/jovr.v14i4.5455

**Published:** 2019-10-24

**Authors:** Hassan Hashemi, Mohammad Saatchi, Abbasali Yekta, Babak Ali, Hadi Ostadimoghaddam, Payam Nabovati, Mohamadreza Aghamirsalim, Mehdi Khabazkhoob

**Affiliations:** ^1^Noor Research Center for Ophthalmic Epidemiology, Noor Eye Hospital, Tehran, Iran; ^2^Noor Ophthalmology Research Center, Noor Eye Hospital, Tehran, Iran; ^3^Department of Optometry, School of Paramedical Sciences, Mashhad University of Medical Sciences, Mashhad, Iran; ^4^Refractive Errors Research Center, Mashhad University of Medical Sciences, Mashhad, Iran; ^5^Rehabilitation Research Center, Department of Optometry, School of Rehabilitation Sciences, Iran University of Medical Sciences, Tehran, Iran; ^6^Eye Research Center, Tehran University of Medical Sciences, Tehran, Iran; ^7^Department of Psychiatric Nursing and Management, School of Nursing and Midwifery, Shahid Beheshti University of Medical Sciences, Tehran, Iran

**Keywords:** Asthenopia, Astigmatism, Photophobia, Prevalence

## Abstract

**Purpose:**

To determine the prevalence of asthenopia and its associated factors in a sample of university students in Iran.

**Methods:**

In this cross-sectional study, participants were selected using multistage cluster sampling. Presence of at least one of the 10 symptoms—foreign body sensation, diplopia, blurred vision, eye swelling, dry eye, eye pain, difficulty in sustaining visual operations, decreased visual acuity, tearing, and photophobia—was considered as asthenopia. Ocular examinations, including uncorrected/corrected visual acuity measurement, objective/subjective refraction, cover test, amplitude of accommodation (AA), and near point of convergence (NPC) were performed.

**Results:**

Of the 1,462 students (mean age: 22.8 ± 3.1 years), 73% were women. The age- and gender-standardized prevalence was 70.9% (95% confidence interval [CI]: 68.3–73.5), 39.8% (95% CI: 36.4–43.1), and 19.7% (95% CI: 16.0–23.3) based on the presence of at least one, two, and three symptoms, respectively. The prevalence was significantly higher in females (*P* = 0.048), hyperopic students (*P* < 0.001), and astigmatic participants (*P* < 0.001). The mean AA and NPC were 9.7 ± 2.6 D and 10.2 ± 4.2 D (*P* = 0.008) and 7.0 ± 2.1 cm and 7.7 ± 3.9 cm (*P* < 0.001) in participants with and without asthenopia, respectively. Multiple regression model revealed age (28–29 years), astigmatism, and NPC as independent associated factors (odds ratios: 3.51, 1.61, and 0.91, respectively).

**Conclusion:**

This study shows relatively high prevalence of asthenopia in university students. Demographic factors and visual system disorders are important risk factors and timely correction of conditions may lead to decreased asthenopia.

##  INTRODUCTION

Asthenopia is a general term used to define a group of somatic or perceptive symptoms that usually occur following computer work, reading, or other near visual activities.^[1]^ Headache, sore and/or itching eyes, blurred vision, epiphora, dry eye, double vision, photophobia, and foreign body sensation are the most common complaints, with several studies considering the presence of at least one of these symptoms as asthenopia.^[2,3]^ Few studies have addressed the prevalence of asthenopia as compared with studies on other ocular conditions and diseases. However, there are reports of a prevalence of 12.4–32.2% in children below 18 years^[4]^ to 57% in students below 30 years.^[5]^ Despite contradictory reports on the causes of asthenopia, epidemiological studies have identified three major groups of risk factors: visual disorders, such as refractive errors and accommodative dysfunctions;^[6,7]^ psychological factors such as daily stress and poor mental state;^[8]^ and environmental factors such as low ambient lighting, nonstandard monitor brightness;^[9]^ and long study periods. Social networking with the resulting near work and increased duration of eye exposure to smartphones, laptop screen, and tablets and educational tasks and long studying hours at the graduate level have made students vulnerable to asthenopic symptoms. Considering the importance of ocular health in the educational success and the interference of asthenopia with visual activities resulting in learning disorders and decreased quality of life, and because no study has already evaluated the prevalence of asthenopia in Iranian university students, the present study was conducted to determine the prevalence of asthenopia and its associated risk factors in a sample of Iranian university students.

##  METHODS

The present university-based, cross-sectional study was conducted in Kazerun, south of Iran, in 2017. Multi-stage sampling was used to select the participants. There are four universities in Kazerun and each university was considered a stratum. Next, a list of all academic majors in each university was prepared, and each academic major was considered a cluster. In each university, a number of majors were randomly selected using a random number table in proportion to their share in the total number of majors of four universities. Finally, a total of 27 majors were selected. After coordinating with the Deputy of Educational Affairs of each university, the list of all students in each major was obtained and each student was assigned a unique code in a continuous manner. Subsequently, proportional to the size of students in each major, some numbers were randomly selected from a table of random numbers. In the next step, students whose unique code matched the last two (or three) digits of the random numbers were selected. Telephone numbers of the selected subjects were obtained from their universities; these students were contacted to invite them to participate in the study after explaining its objectives.

Refractive error was considered as the main outcome of the study. The prevalence of myopia was selected to reach a maximum sample size. Based on similar studies^[10,11]^ and considering a prevalence of 41%, type I error of 0.05, precision of 0.04, and a sample size of 580 were estimated. With regard to the sampling method, a design effect of 2.5 was considered. After an addition of 10% non-response rate to the calculated sample size, the final sample size was 1,595 participants.

###  Examinations

First, uncorrected visual acuity (UCVA) was measured using Snellen eye chart at 6 meters(m). Following this, objective refraction was calculated using the auto-refractometer (Topcon RM-8800; Topcon Corp., Tokyo, Japan), and the results were refined using the Heine Beta 200 retinoscope (Heine Optotechnik; Herrsching, Germany). Next, subjective refraction was used to determine the best optical correction, and the best corrected distance and near visual acuities (BCVAs) were recorded.

In the next stage, binocular and accommodative examinations were performed according to the best optical correction. First, unilateral and alternate cover tests were conducted at 6 m and 40 cm, following which the magnitude of near and distance phoria was measured using the alternate cover test and prism bar. An accommodative target was used for the cover test, including one line above the BCVA on the near and distance Snellen charts. In the next stage, Dander's push-up method was used to monocularly measure the near point of accommodation (NPA) using the Royal Air Force Rule (RAF), with a line equivalent to the visual acuity of 20/25 as the accommodative target. The NPA was subsequently converted to accommodative amplitude (AA) in diopters (D) by dividing 100 by NPA. Near point of convergence (NPC) in cm was then measured by slowly moving the accommodative target (a character one line above the BCVA) toward the participant's eyes along the midline until the participant reported diplopia or the examiner observed fusion break. After completion of optometric examinations, cyclo-refraction was performed by instilling two drops of 1% cyclopentolate, separated by 5 min and repeating retinoscopy 30 min after the last drop.

###  Definition of Asthenopia

To be consistent with other studies, the presence of at least one of the ten symptoms—foreign body sensation, diplopia, blurred vision, eye swelling, dry eye, eye pain, difficulty in sustaining visual operations, decreased visual acuity, tearing, and photophobia—occurring during near visual activities was considered as asthenopia. Moreover, the prevalence of asthenopia was determined based on the presence of two or three symptoms. The exclusion criteria included age > 40 years; unwillingness to participate in the study; history of intraocular surgery and ocular trauma; systemic conditions or diseases affecting accommodation and binocular vision including hormonal or metabolic diseases and conditions such as pregnancy, diabetes, and thyroid dysfunctions, and neurologic diseases such as myasthenia gravis and multiple sclerosis; the use of ocular or systemic medications affecting accommodation and binocular vision including cycloplegic drops, central nervous system stimulants, and phenothiazine derivates; strabismus, amblyopia; and BCVA < 20/40 in either eye.

###  Statistical Analysis

The Stata software version 11 (StataCorp; College Station, TX, USA) was used for data analysis. The prevalence of asthenopia was reported as percentage and 95% confidence interval (CI). To determine the associated risk factors of asthenopia, multiple logistic regression was used in a backward manner by running the survey analysis command of Stata according to the presence of at least one symptom from the aforementioned asthenopic symptoms. The age- and gender-standardized prevalence of asthenopia was calculated based on the age and gender distribution of students in 2015 using direct standardization.

Variables evaluated in this study included age, gender, body mass index (BMI), years of study, anisometropia, astigmatism, spherical equivalent (SE) of refraction, near phoria, AA, and NPC. To determine the years of study and its effect on asthenopia, students were divided into two groups: < 2 years (four academic terms) and > 2 years. According to the World Health Organization (WHO) guidelines,^[12]^ BMI was categorized as underweight (< 18.5), normal (18.5–24.5), and overweight (< 24.5). Based on cycloplegic refraction, myopia and hyperopia were defined as ≤ 0.50 and > 0.50 D of SE, respectively. Anisometropia was defined as SE difference ≥ 1.00 D between the eyes. Due to the significant correlation of both eyes in AA (Pearson's correlation coefficient = 0.97), only the AA of the right eye was considered for statistical analysis. *P* < 0.05 was considered statistically significant.

###  Ethical Considerations

The Ethics Committee of the Mashhad University of Medical Sciences approved the protocol of the study according to the Declaration of Helsinki. Informed consent was obtained from all participants. The students were assured that their data would remain anonymous and confidential.

##  RESULTS

**Figure 1:**
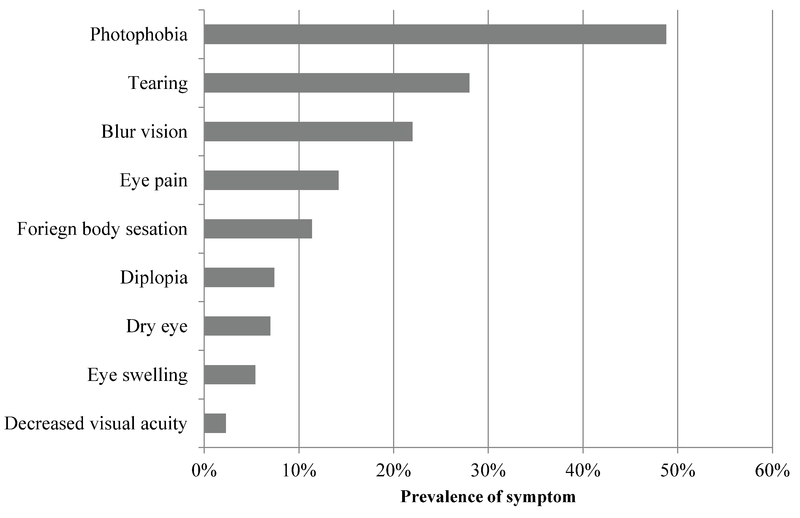
The prevalence of asthenopic symptoms in 1,465 students above 18 years in the city of Kazerun.

Of the 1,595 invited individuals, 1,462 participated in the study. Of these selected students with a mean age of 22.8 ± 3.1 years (range: 18–40 years), 73% were women. Based on the presence of at least one, two, and three symptoms, the prevalence of asthenopia calculated to be 71.2% (95% CI: 68.4–74.0), 40.6% (95% CI: 37.7–43.4), and 19.7% (95% CI: 16.3%–23.2%), respectively. Table 1 presents the prevalence of asthenopia based on one, two, and three symptoms according to gender, age, SE, astigmatism, BMI, academic term, near phoria, and anisometropia. As shown in Table 1, the prevalence of asthenopia was significantly higher in females (*P* = 0.048), hyperopic students (*P* < 0.001), and participants with astigmatism (*P* < 0.001). The mean AA was 9.7 ± 2.6 D and 10.2 ± 4.2 D in participants with and without asthenopia, respectively (*P* = 0.008). The mean NPC was 7.0 ± 2.1 cm and 7.7 ± 3.9 cm in asthenopic and non-asthenopic students, respectively (*P* < 0.001). Figure 1 shows the prevalence of asthenopic symptoms: photophobia was the most common symptom (48.7%). Table 2 presents the prevalence of asthenopic symptoms by gender. According to Table 2, the prevalence of most of the asthenopic symptoms was higher in females than in the male students. Table 3 presents the results of logistic regression model for associated risk factors of asthenopia. As seen in multiple regression model, age group of 28 to 29 years, astigmatism, and NPC were independent associated risk factors of asthenopia with odds ratios of 3.51, 1.61, and 0.91, respectively.

**Table 1:** The prevalence of asthenopia among 1,462 participants


	**Number of Symptoms**				**Total Number of Subjects**
	**One or more**	**P-Value**	**Two or more**	**Three or more**	
	**Percent (95% CI)**	**Percent (95% CI)**	**Percent (95% CI)**	
**Age sex standardized**	70.9 (68.3–73.5)	39.8 (36.4–43.1)	19.7 (16.0–23.3)	
**Sex**	Male	65.5 (60.8–70.2)	0.048	31.6 (26.7–36.5)	12.8 (9.8–15.8)	389
	Female	73.3 (70.5–76.1)	43.8 (41.1–46.6)	22.2 (18.6–25.9)	1,073
**Age group (year)**	18–19	72.2 (66.9–77.9)	0.219	38.9 (30.5–47.3)	23.3 (17.6–29.1)	90
	20–21	70.2 (65.5–75.0)	37.2 (32.3–42.1)	19.3 (13.5–25.0)	441
	22–23	71.6 (67.4–75.8)	42.3 (38.2–46.4)	18.5 (13.8–23.2)	546
	24–25	72.6 (66.8–79.4)	42.4 (35.7–49.2)	22.4 (16.4–28.5)	205
	26–27	75.3 (64.8–84.8)	48.1 (31.6–64.5)	23.4 (11.5-35.3)	77
	28–29	81.5 (66.2–96.9)	55.3 (41.2–69.3)	23.7 (14.9–32.5)	38
	≥ 30	58.4 (45.0–71.8)	29.2 (16.5–41.9)	13.8 (4.5–23.2)	65
**Refractive errors**	Emmetropia	70.2 (62.9–71.0)	<0.001	34.3 (29.5–32.9)	15.5 (10.5–20.5)	803
	Hyperopia	79.6 (73.9–78.7)	47.5 (44.8–50.1)	24.6 (20.3–28.9)	625
	Myopia	79.4 (53.7–99.9)	61.7 (35.4–88.0)	29.4 (8.2–50.5)	34
**Astigmatism**	No	68.5 (65.9–71.1)	<0.001	36.7 (33.8–39.7)	16.9 (12.8–21.0)	1,030
	Yes	77.7 (73.0–82.4)	49.7 (45.0–54.4)	26.3 (23.2–29.5)	432
**BMI**	Underweight	72.7 (64.2–81.3)	0.774	40.8 (28.5–53.1)	21.0 (9.4–32.7)	147
	Normal	70.6 (66.9–74.4)	40.3 (38.2–42.4)	19.1 (16.2–22.0)	986
	Overweight	72.3 (68.2–76.4)	41.6 (34.9–48.4)	20.9 (16.3–25.5)	329
**Semester**	1–4 (≤ 2 year)	72.4 (68.3–76.5)	0.374	39.3 (35.5–43.1)	20.0 (15.3–24.7)	709
	> 4 (> 2 year)	70.3 (66.7–73.8)	41.8 (38.2–45.4)	19.5 (14.4–24.5)	753
**Near phoria**	No	70.5 (66.4–74.7)	0.141	40.4 (37.5–43.3)	19.2 (15.9–22.5)	974
	Exo	71.7 (68.3–75.2)	39.8 (36.7–43.0)	19.2 (13.8–24-7)	467
	Eso	86.8 (74.2–99.5)	60.5 (43.7–77.3)	34.9 (27.7–51.1)	38
	Hyper	57.1 (18.5–95.8)	14.3(8.2–46.8)	14.2 (8.2–46.7)	7
**Anisometropia**	No	70.9 (68.1–73.7)	0.283	40.0 (37.3–42.8)	19.3 (16.2–22.5)	1,409
	Yes	79.2 (63.3–95.1)	54.7 (34.7–74.4)	30.1 (16.2–44.1)	53
	
	
The presence of at least one of the 10 symptoms of foreign body sensation, diplopia, blurred vision, eye swelling, dry eye, eye pain, difficulty in sustaining visual operations, decreased visual acuity, tearing, and photophobia was considered as asthenopia.						
BMI, body mass index; CI, confidence interval; Eso, esophoria; Exo, exophoria						

**Table 2:** Prevalence of symptoms by gender


**Symptom**	**Female (95% CI)**	**Male (95% CI)**	***P*** **-value**
**Eye pain**	15.4 (12.4–18.5)	10.5 (7.8–13.2)	0.017
**Dry eye**	7.5 (5.3–9.7)	5.6 (3.7–7.5)	0.211
**Eye swelling**	6.4 (4.2–8.5)	2.5 (0.9–4.1)	0.004
**Blurred vision**	23.6 (19.0–28.2)	17.4 (14.2–20.7)	0.012
**Diplopia**	8.39 (6.0–10.7)	4.6 (2.9–6.3)	0.015
**Foreign body sensation**	11.4 (8.7–14.2)	11.0 (7.9–14.2)	0.827
**Photophobia **	51.8 (48.3–55.3)	40.3 (32.3–48.3)	< 0.001
**Tearing **	30.0 (25.2–34.7)	22.6 (16.5–28.7)	0.005
**Decreased visual acuity**	2.1 (0.8–3.4)	2.5 (1.4–3.7)	0.627
CI, confidence interval						

**Table 3:** Simple and multi-variable logistic regression for the associated risk factors of asthenopia


	**OR Unadjusted**	**95% CI**	**P-Value**	**OR Adjusted**	**95% CI**	**P-Value**
**Sex**			
Female	1.44	1.01–2.05	0.041		
Male	Reference			
**Age group (year)**			
18–19	1.85	0.85–4.02	0.111	1.66	0.73–3.77	0.203
20–21	1.68	0.89–3.18	0.101	1.68	0.78–3.65	0.167
22–23	1.79	1.03–3.13	0.042	1.69	0.87–3.29	0.108
24–25	1.89	0.90–3.96	0.085	1.89	0.83–4.31	0.116
26–27	2.17	0.90–5.20	0.077	2.06	0.93–4.55	0.071
28–29	3.15	1.40–7.07	0.01	3.51	1.26–9.89	0.021
≥ 30	Reference		Reference	
**Refractive error**			
Emmetropia	Reference			
Myopia	1.58	1.27–1.98	0.001		
Hyperopia	1.89	0.38–9.35	0.394		
**Astigmatism**			
No	Reference		Reference	
Yes	1.6	1.24–2.06	0.002	1.61	1.22–2.13	0.003
**BMI**			
Normal	Reference			
Underweight	1.1	0.71–1.72	0.617		
Overweight	1.08	079–1.48	0.584		
**Semester**			
1–4(≤ 2 year)	Reference			
> 4(> 2 year)	0.9	0.69–1.17	0.418		
**Anisometropia**			
No	Reference			
Yes	1.58	0.584.14	0.337		
**Near Phoria**			
No	Reference			
Eso	1.06	0.79–1.42	0.671		
Exo	2.76	0.98–7.75	0.054		
Hyper	0.56	0.12–2.66	0.427		
AA (diopter)	0.95	0.92–0.98	0.007		
NPC (cm)	0.91	0.87–0.96	< 0.002	0.91	0.86–0.97	0.008
	
	
AA, accommodative amplitude; BMI, body mass index; NPC, near point of convergence; OR, odds ratio						

##  Discussion

The present study is the first of its kind to demonstrate the prevalence of asthenopia and its associated risk factors in students above 18 years of age in Kazerun, south of Iran. According to our findings and based on the presence of at least one symptom, the prevalence of asthenopia was calculated to be 71%, which is much higher than the results of a similar study (57%) conducted by Han et al in Chinese students with a mean age of 21 years.^[5]^ Moreover, another study showed a prevalence of 46% for asthenopia in Indian computer operators with a mean age of 25 years.^[1]^ Aakre et al^[10]^ and Ostrovsky et al^[11]^ evaluated the prevalence of asthenopia in regular computer users. The limited number of population-based studies, especially studies on students aged 18 to 30 years, makes it difficult to compare our results with similar studies. In other words, lack of studies on similar age groups; use of different criteria and outcomes, including eye strain and fatigue,^[13]^ and evaluation of some occupations with more exposure to computers and monitors hinder the comparison of the results of the present study with other similar studies. Our findings showed that based on at least two or three symptoms, the prevalence of asthenopia was much higher than that in the younger age groups in other studies (adolescents under 18 years). In a study by Hashemi et al,^[14]^ the prevalence of asthenopia based on at least two symptoms was 24% in adolescents aged 12 to 18 years, whereas our findings revealed a prevalence of 40% in the university students. Considering the importance of students in individual and social achievements, timely diagnosis and treatment of this condition are essential. According to the conducted studies, adequate sleep, regular intake of vegetables, and a good mental frame play a crucial role in preventing asthenopia.^[5,15]^ Therefore, considering the lifestyle of Iranian students, their life in dormitories and lack of proper nutrition, a high prevalence of asthenopia is not unexpected in this group. Another point related to the high prevalence of asthenopia is that given the various symptoms considered for its diagnosis, each symptom may be associated with another disease or condition; for example, headache is one of the most common symptoms in several diseases.[< xref  ref - type =" bibr " rid ="B16">16</ xref >] According to our findings, photophobia was the most common symptom in more than 48% of the cases, which is not consistent with the results of some studies that reported headache or tearing as the most common symptom of asthenopia.^[4,17]^ There exist several reasons for the sensitivity of eye to light; however, an underlying disease can also cause photophobia. Moreover, ocular surface diseases may result in photophobia.^[18]^ The high prevalence of photophobia in our study population is an alarm sign and warrants further investigation into its underlying reasons. Based on these findings, the prevalence of asthenopia in women was around 8% and more than in men.

There are contradictory results on the effect of gender; moreover, some studies have failed to find a significant effect of gender on asthenopia.^[5,19]^ Contrary to our results, Han et al^[5]^ and Agrawal et al^[10]^ found no significant difference in the prevalence of asthenopia between men and women, whereas Shima et al^[20]^ and Bahanderi et al^[1]^ reported a higher prevalence of asthenopia symptoms in women. Furthermore, except for foreign body sensation, dry eye, and decreased visual acuity, the prevalence of other symptoms was higher in women. Different physiological structure and pain threshold as well as different lifestyles of boys and girls may significantly affect the odds of developing asthenopia and asthenopic complaints. Our findings showed that the prevalence and odds of asthenopia increased significantly from 20 to 29 years but decreased thereafter. Moreover, Bahanderi et al^[1]^ and Maccoi et al^[8]^ have reported aging as a determinant of asthenopia. Several studies have confirmed age as one of the most important determinants of different ocular disorders. However, it is reported that in addition to age,^[21,22]^ the duration of computer use greatly affects the development of asthenopia. The bachelor's level study is usually finished by 22 years of age, following which students start post-graduate courses. Long years of academic studies and increased exposure to computers could explain the increased prevalence of asthenopia in subjects below 30 years. Furthermore, the study population has an important role in explaining the relationship between age and the prevalence of asthenopia. For example, in a population-based study by Schelini et al,^[23]^ the highest prevalence of asthenopia was seen in the first two decades of life and its prevalence decreased significantly after the age of 40 years. Therefore, taking into consideration the repeated computer work and reading, a higher prevalence of asthenopia is expected in university students and those in academia.

Based on the available reports, the refractive status, especially astigmatism, is a crucial factor in developing asthenopia.^[6,19,24]^ Our findings showed that odds of asthenopia were 1.61 times higher in astigmatic subjects as compared with that in students without astigmatism. Moreover, a population-based study in Brazil showed that astigmatism was the most important risk factor associated with asthenopia.^[23]^ Similarly, Kotegava et al^[25]^ reported that proper and adequate correction of refractive errors decreased the prevalence of asthenopia and improved accommodative dynamics in the study population. Similar results were reported by Abdi et al.^[26]^

Our findings revealed no significant association between asthenopia and phoria. Kaufmann et al^[27]^ reported that it was difficult to draw a causal relationship between phoria and asthenopia for three reasons: lack of objective criteria for detecting asthenopia, non-recognition of the pathogenetic mechanism of the effect of phoria on asthenopia, and the presence of other conditions with similar symptoms such as dry eye, accommodative anomalies, and aniseikonia.

Results obtained from most studies suggest that prolonged computer work is an important risk factor in the development of asthenopia. For instance, Han et al^[5]^ reported that the odds of asthenopia were 21% higher in students who worked on computer every day as compared with those without daily use of computer. Moreover, it has been reported that computer work for 6 h a day or 30 h a week has a strong association with asthenopia.

One of the limitations of this study was that we did not evaluate near work duration, which we plan to consider in future studies. Moreover, the possible organic causes of asthenopia were not assessed. Although exophoria is a well-known determinant of asthenopia, its effect was not significant in our study, probably owing to the low number of participants suffering from this type of phoria.

In conclusion, the present study demonstrated for the first time a high prevalence of asthenopia in Iranian university students above 18 years as compared with the results of other similar studies, including those on subjects below 18 years.

##  Financial Support and Sponsorship

This project was supported by Mashhad University of Medical Sciences.

##  Conflicts of Interest

There is no conflict of interest.
